# Sphingosine Promotes Embryo Biomass in Upland Cotton: A Biochemical and Transcriptomic Analysis

**DOI:** 10.3390/biom11040525

**Published:** 2021-04-01

**Authors:** Li Wang, Xiaodong Suo, Yujie Liu, Chen Liu, Ming Luo

**Affiliations:** 1 Zhengzhou Research Base, State Key Laboratory of Cotton Biology, Zhengzhou University, Zhengzhou 450001, China; wangli07-2@163.com (L.W.); liuyujie19@126.com (Y.L.); liuchen_lc@163.com (C.L.); 2 State Key Laboratory of Cotton Biology, Institute of Cotton Research, Chinese Academy of Agricultural Sciences, Anyang 455000, China; 3 Key Laboratory of Biotechnology and Crop Quality Improvement of Ministry of Agriculture, Biotechnology Research Center, Southwest University, Chongqing 400716, China; sxd18883770582@163.com

**Keywords:** cotton, embryo growth, sphingolipids, PHS, transcriptomic analysis

## Abstract

Sphingolipids are essential membrane components and signal molecules, but their regulatory role in cotton embryo growth is largely unclear. In this study, we evaluated the effects of treatment with the sphingolipid synthesis inhibitor fumonisin B1 (FB1), the serine palmityl transferase (SPT) inhibitor myriocin, the SPT sphingolipid product DHS (d18:0 dihydrosphingosine), and the post-hydroxylation DHS product PHS (t18:0 phytosphingosine) on embryo growth in culture, and performed comparative transcriptomic analysis on control and PHS-treated samples. We found that FB1 could inhibit cotton embryo development. At the five-day ovule/embryo developmental stage, PHS was the most abundant sphingolipid. An SPT enzyme inhibitor reduced the fresh weight of embryos, while PHS had the opposite effect. The transcriptomic analysis identified 2769 differentially expressed genes (1983 upregulated and 786 downregulated) in the PHS samples. A large number of transcription factors were highly upregulated, such as zinc finger, *MYB*, *NAC*, *bHLH*, *WRKY*, *MADS*, and *GRF* in PHS-treated samples compared to controls. The lipid metabolism and plant hormone (auxin, brassinosteroid, and zeatin) related genes were also altered. Our findings provide target metabolites and genes for cotton seed improvement.

## 1. Introduction

Crop seeds are not only an important source of human food, animal feed, and industrial raw materials, but are also the starting point for plant growth and development, organ yield, quality, and environmental adaptability. Cotton is not only the most important fiber crop, but also an important oil crop, and cottonseed meal also contains many types of high quality proteins. Seed weight is an important crop trait, and is regulated by many genetic factors. Seed size varies considerably among different plant species. It is also regulated by both endogenous signals and developmental conditions within any one species. Therefore, the molecular regulation mechanism of seed formation is a very important developmental biology issue. Cotton seeds, which develop from fertilized embryos, are not only reproductive organs, but also have important economic value. However, the development of cotton embryos remains poorly understood.

Lipid rafts are microregions containing special lipids and proteins, located in the membrane lipid bilayer, and are mainly composed of sphingolipids, sterols, and proteins [1]. Lipid rafts are considered to be an important part of biomembrane, and contain many of the components responsible for biomembrane properties [2]. Sphingolipids, not only as membrane components, but also as signal molecules, play an important role in various plant life processes. The functions of sphingolipids in plants have been gradually revealed by studies of Arabidopsis gene mutations related to sphingolipid synthesis and metabolism. For example, in the *sbh1*/*sbh2* mutant, t18:0 (phytosphingosine, PHS) cannot be detected, but d18:0 (dihydrosphingosine, DHS) and d18:1 accumulate heavily. This double mutant is a dwarf, with inhibited cell elongation and division, and constitutionally up-regulated hypersensitivity and programmed cell death-related genes [3]. In *sld1*/*sld2* mutants, Δ8 desaturation of the component long-chain bases (LCBs) is undetectable, which produces no significant phenotypic change under normal conditions, but the rates of yellowing and apoptosis are faster at low temperatures (0 °C) [4]. The mutation *loh1/loh3* is lethal, resulting in substantial accumulation of long-chain sphingolipids and reduction of very-long-acyl-chain sphingolipids [5]. These studies reveal that plant sphingolipids are functionally complex in plant growth and development.

In a previous study, a total of 95 sphingolipids were detected in cotton fibers and embryos from in vitro ovule culture, including six major categories of sphingolipids, namely, LCB, LCB-1-phosphates (LCB-1P), ceramides (Cer), hydroxyceramides (hCer), glucosylceramides (GluCer), and glycosyl inositol phosphoceramides (GIPC) [6]. In cotton fibers and embryos treated with the sphingolipid synthesis inhibitor fumonisin B1 (FB1), most simple sphingolipids were significantly increased, while most complex sphingolipids were significantly decreased. Moreover, FB1 leads to severe impairment of fiber elongation [6]. In this study, we aimed to increase understanding of the impact of sphingolipids in embryo development, hypothesizing that they were significant factors. We found that FB1 could block cotton embryo growth. We determined the sphingolipid content of cotton ovules/embryos at different developmental stages and found that PHS was the most abundant sphingolipid. PHS is the hydroxylated product of DHS [7]. The serine palmityl transferase (SPT) is responsible for de novo synthesis of DHS [7]. Therefore, we selected SPT inhibitor myriocin and PHS to further elucidate the function of sphingosine in embryo development. Myriocin was found to inhibit embryo growth, while PHS increased embryo fresh weight. Furthermore, we performed transcriptomic analysis of embryos treated with PHS to identify relevant key biological pathways in cotton embryo growth. Our results provide new insights into the regulatory mechanisms behind cotton embryo growth.

## 2. Materials and Methods

### 2.1. Cotton Materials and In Vitro Ovule Culture

The upland cotton variety *Gossypium hirsutum* L. acc. TM-1 used in this study was grown in the field in Zhengzhou, Henan Province. Cotton bolls tagged and dated at anthesis were collected two days post-anthesis (DPA) and sterilized with mercuric chloride (0.1% aqueous solution). The sterilized embryos were incubated in Beasley and Ting’s medium [8] at 32 °C in the dark for either 5 days or 10 days. Within each duration group, one of the following was added to each culture container: FB1 (1 µM), myriocin (0.2 µM, 1 µM, or 2 µM), DHS (2 µM or 6 µM), or PHS (2 µM, 6 µM, or 20 µM). They were purchased from Sigma-Aldrich (St. Louis, MO, USA) and first dissolved in DMSO (Amresco, Washington, DC, USA), and the final concentration of DMSO in the medium was 0.2% (*v*/*v*). Six µM PHS-treated and control samples cultured for 10 days were collected for RNA extraction.

### 2.2. Lipid Extraction and Lipidomics

Ovules/embryos (without fiber) collected from cotton bolls at anthesis and after 5, 10, 15, and 20 days of development were placed in liquid nitrogen and kept at −80 °C. After sample collection was completed, lipid extraction and lipidomic analysis were performed by the Lipidall Technologies Company Limited (http://www.lipidall.com/, accessed on 22 June 2019), as described previously [6,9,10,11].

### 2.3. RNA-Sequencing (RNA-seq)

Total RNA from control and PHS samples was extracted using the RNAprep Pure Plant Kit (Tiangen, Beijing, China) according to the manufacturer’s protocol. RNA quality was measured using an Agilent 2100 Bioanalyzer (Agilent Technologies, Palo Alto, CA, USA) and RNase-free agarose gel electrophoresis. Libraries were prepared using the NEB Next^®^ Ultra™ RNA Library Prep Kit for Illumina^®^ (#E7530L; New England Biolabs, Ipswich, MA, USA) according to the manufacturer’s protocol. Briefly, enrichment of mRNA was carried out using Oligo (dT) beads. The mRNA was broken into short fragments after total RNA extraction, and then reverse transcribed into cDNA using random primers. Second-strand cDNA was synthesized using DNA polymerase I, RNase H, dNTP, and buffer. The cDNA fragments were then purified with a QIAquick PCR Extraction Kit (Qiagen, Venlo, The Netherlands). Finally, they were end-repaired, an A base was added, and the resulting fragments were ligated to Illumina (San Diego, CA, USA) sequencing adapters. The ligation products were size selected by agarose gel electrophoresis, amplified by PCR, and sequenced using the Illumina Novaseq6000 by Gene Denovo Biotechnology Co. (Guangzhou, China). RNA-Seq analyses were performed on three independent biological replicates.

### 2.4. Bioinformatic Analysis

Reads obtained from the sequencing machines were filtered by fastp (version 0.18.0) to remove raw reads containing adapters or low-quality bases and obtain high-quality clean reads [12]. Reads were mapped to the ribosome RNA database using the short reads alignment tool Bowtie2 (version 2.2.8) [13] and then removed to generate clean reads for assembly and gene abundance calculation.

HISAT2. 2.4 [14] with “-rna-strandness RF” was used to map paired-end clean reads to the reference *Gossypium hirsutum* L. acc. TM-1 genome, which is available at http://ibi.zju.edu.cn/cotton (accessed on 10 December 2020) [15]. StringTie v1.3.1 was used to assemble the mapped reads of each sample [16,17] using a reference-based approach. The fragment per kilobase of transcript per million mapped reads (FPKM) value was calculated to quantify its expression abundance using RSEM [18]. DESeq2 was used for RNA differential expression analysis between control and PHS samples [19]. Genes with a false discovery rate (FDR) < 0.05 and absolute fold change ≥2 were considered to be significantly differentially expressed genes.

The Gene Ontology (GO) database [20] was used to analyze the biological significance and assess the functionality of genes differentially expressed between control and PHS samples. GO has three ontologies: Molecular function, cellular component, and biological process. The basic unit of GO is GO-term. Each GO-term belongs to a type of ontology. GO enrichment analysis provides all GO terms that significantly enriched in DEGs comparing to the genome background, and filter the DEGs that correspond to biological functions. Firstly, all DEGs were mapped to GO terms in the Gene Ontology database (http://www.geneontology.org/, accessed on 15 December 2020), gene numbers were calculated for every term, significantly enriched GO terms in DEGs comparing to the genome background were defined by hypergeometric test. The calculating formula of *p*-value is:

(1) $$P=1-\overset{m-1}{\underset{i=0}{\sum }}\frac{\left(\begin{array}{c} M\\ i \end{array}\right)\left(\begin{array}{c} N-M\\ n-i \end{array}\right)}{\begin{array}{c} N\\ n \end{array}}$$

Here, *N* is the number of all genes with GO annotation; *n* is the number of DEGs in *N*; *M* is the number of all genes that are annotated to the certain GO terms; *m* is the number of DEGs in *M*. The calculated *p*-value were gone through FDR Correction, taking FDR ≤ 0.05 as a threshold. GO terms meeting this condition were defined as significantly enriched GO terms in DEGs. This analysis was able to recognize the main biological functions that DEGs exercise.

Biological pathways associated with DEGs were analyzed using the Kyoto Encyclopedia of Genes and Genomes (KEGG) pathway databases [21]. Pathway enrichment analysis identified significantly enriched metabolic pathways or signal transduction pathways in DEGs comparing with the whole genome background. The calculating formula is the same as that in GO analysis.

(2) $$P=1-\overset{m-1}{\underset{i=0}{\sum }}\frac{\left(\begin{array}{c} M\\ i \end{array}\right)\left(\begin{array}{c} N-M\\ n-i \end{array}\right)}{\begin{array}{c} N\\ n \end{array}}$$

Here, *N* is the number of all genes that, with KEGG annotation, *n* is the number of DEGs in *N*, *M* is the number of all genes annotated to specific pathways, and *m* is number of DEGs in *M*. The calculated *p*-value was gone through FDR Correction, taking FDR ≤ 0.05 as a threshold. Pathways meeting this condition were defined as significantly enriched pathways in differentially expressed genes (DEGs).

### 2.5. Heat Map Drawing

RNA-seq data for the DEGs identified above were used for heat map analysis. The FPKM (fragments per kilobase of transcript per million fragments mapped) values of differentially expressed genes related to transcription factors, lipid metabolism, and plant hormones between control and PHS samples were visualized with heat-maps generated by v3.5.1 of R (https://CRAN.Rproject.org/package=pheatmap, accessed on 7 June 2020). All the pictures in the text were sheared by adobe photoshop CS6 software and then plotted to figures using adobe illustrator CS6.

### 2.6. Semi-Quantitative PCR

First-strand cDNAs were synthesized using the Prime Script™ RT Reagent Kit with gDNA Eraser (Takara, Kyoto, Japan). Semi-quantitative PCR reactions were performed using 2xTaq Plus Master Mix (Dye Plus) (Vazyme, Nanjing, China). The PCR conditions were as described previously [6]. Three biological repetitions were performed. The specific primers for the selected genes and the internal control (*Gossypium hirsutum polyubiquitin protein* gene, *Gbp*) are listed in Table S1.

## 3. Results

### 3.1. Sphingolipid Synthesis Inhibitor Reduced Cotton Embryo Biomass

**Figure 1:**
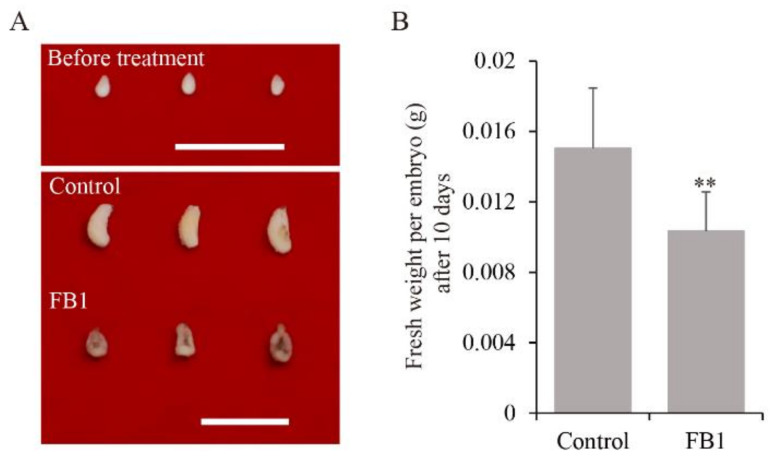
Sphingolipid synthesis inhibitor inhibited cotton embryo growth. (**A**) Phenotype of 2 DPA embryo before treatment and FB1 treated embryos after 10 days of in vitro culture. Scale bars = 1 cm. (**B**) Fresh weight of FB1-treated cultured embryos after 10 days. Values represent means ± SD (*n* = 30). ** indicates *p* ≤ 0.01.

In this study, we observed that FB1 reduced embryo fresh weight largely by an in vitro cotton ovule culture system (Figure 1A). We measured the fresh weight of embryos cultured for 10 days and found that the fresh weight of embryos cultured on medium with FB1 were reduced by 31% compared to that of the control (Figure 1B). The results indicated that sphingolipid homeostasis plays an important role in embryo growth.

### 3.2. Sphingosine Is the Main Sphingolipid Component in Upland Cotton Ovules/Embryos

**Figure 2:**
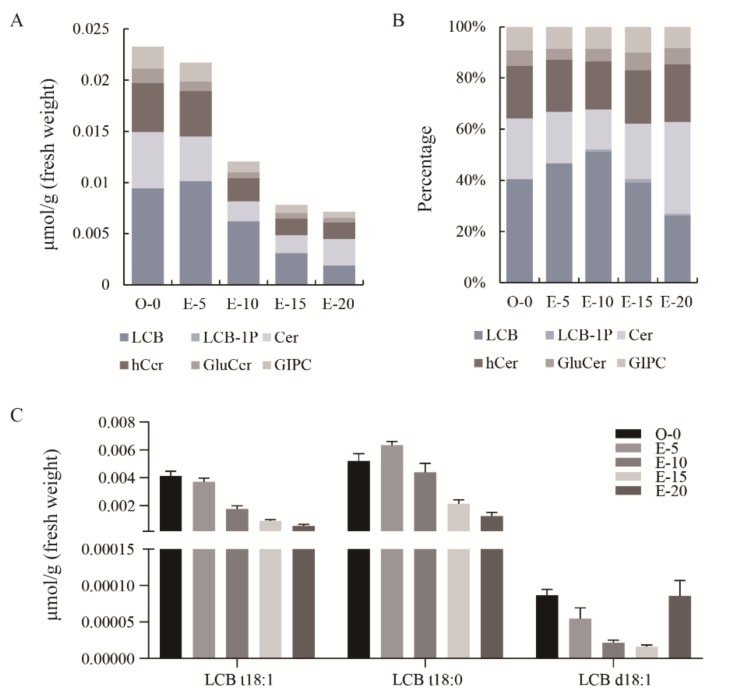
Sphingolipid composition in field-grown upland cotton ovules/embryos at different developmental stages. (**A**) The total content of six major sphingolipid categories in cotton ovules/embryos at five different developmental stages. (**B**) Percentages of the six major categories of sphingolipids in cotton ovules/embryos at different developmental stages. (**C**) Individual sphingosine content of cotton ovules/embryos at different developmental stages. LCB, long chain base; LCB-1P, LCB-1-phosphates; Cer, ceramides; hCer, hydroxyceramides; GluCer, glucosylceramides; GIPC, glycosyl inositol phosphoceramides. O-0 indicates ovules at anthesis. E-5, E-10, E-15, and E-20 indicate embryos 5, 10, 15, and 20 days post anthesis, respectively. Values represent means ± SD (*n* = 3).

We determined the ovule/embryo sphingolipid content at five developmental stages by liquid chromatography and mass spectrometry. Six major categories of sphingolipids were detected in the cotton ovules/embryos, including LCB, LCB-1P, Cer, hCer, GluCer, and GIPC (Table S2). We found that the sphingolipid content in ovules/embryos gradually reduced as the ovules/embryos developed, from 0.02328 µmol/g in ovules at 0 DPA to 0.00714 µmol/g in embryos at 20 DPA, a decrease of 69.30% (Figure 2A). Among the six major categories of sphingolipids, LCB was the most abundant at the first four developmental stages, accounting for more than 39% of the sphingolipids present (Figure 2B). Three sphingosine molecules (LCB t18:1, t18:0 [PHS], and d18:1) were detected in cotton ovules/embryos (Figure 2C). Among them, LCB t18:0 was most abundant at five developmental stages (Figure 2C). These results indicate that sphingosine is the main sphingolipid component in cotton ovules/embryos and may play important roles in ovule/embryo growth.

### 3.3. SPT Enzyme Inhibitor Reduced Cotton Embryo Biomass

**Figure 3:**
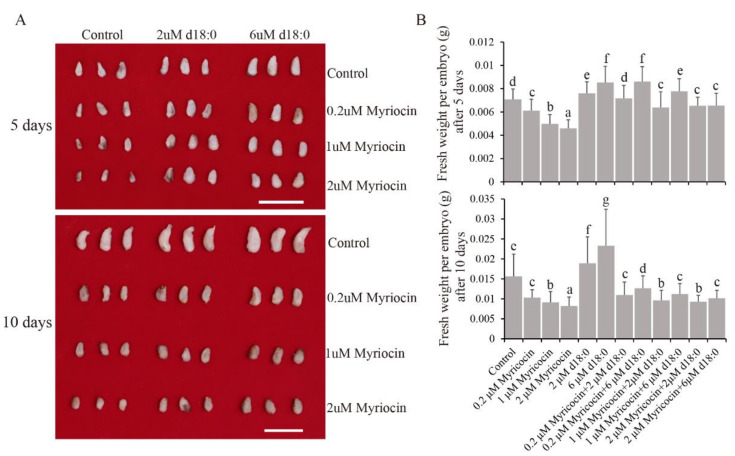
DHS (d18:0 dihydrosphingosine) partially restored the inhibitory effect of myriocin on embryo fresh weight. (**A**) Phenotypic characteristics of myriocin- and DHS-treated embryos after 5 and 10 days of in vitro culture at different concentrations. Scale bars = 1 cm. (**B**) Fresh weight of myriocin- and DHS-treated embryos after 5 days (upper panel) and 10 days (lower panel) at different concentrations. Values represent means ± SD (*n* = 24). Different letters above the bars indicate significant differences at the *p* = 0.05 level.

We treated embryos with three myriocin concentrations (0.2 µM, 1 µM, and 2 µM), and found that myriocin inhibited embryo fresh weight in a dose-dependent manner. Myriocin significantly inhibited embryo growth even at low concentration (0.2 µM); at a dose of 2 µm, the fresh weight of treated embryos was 35.0% and 47.4% lower than that of controls, after 5 and 10 days, respectively (Figure 3). LCB d18:0 was the simplest sphingosine and a downstream product of SPT enzyme activity, so we added DHS to counter the inhibition of embryos by myriocin. The results showed that DHS-treated (2 µM) embryos had significantly higher fresh weights than controls after 5 and 10 days, at 7.4% and 20.6%, respectively (Figure 3). Moreover, at 6 µM DHS, the embryo fresh weights after 5 and 10 days were 20.5% and 48.7% higher, respectively, compared to that of controls (Figure 3). In addition, after 5 days, DHS restored embryo growth in the presence of myriocin at lower concentrations (0.2 µM and 1 µM) and partly restored it at the higher myriocin concentration (2 µM) (Figure 3). The inhibition of embryos treated with myriocin for 10 days was only partially restored by DHS (Figure 3). These results indicated that decreasing SPT enzyme activity could inhibit embryo growth, while exogenous DHS could promote it.

### 3.4. PHS Promoted Cotton Embryo Biomass

**Figure 4:**
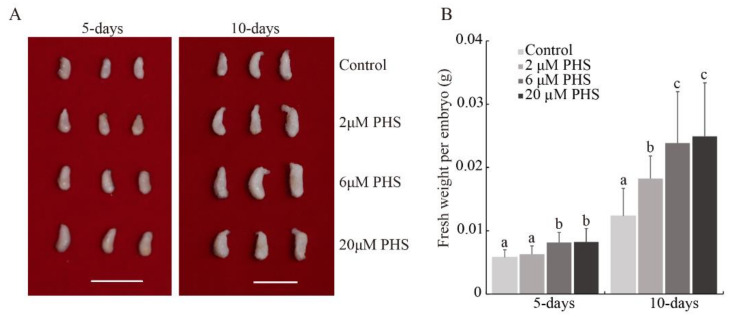
PHS (t18:0, phytosphingosine) increased embryo fresh weight. (**A**) Phenotype of PHS treated embryos after 5 and 10 days at different concentrations. Scale bars = 1 cm. (**B**) Fresh weight of PHS-treated embryos after 5 and 10 days at different concentrations. Values represent means ± SD (*n* ≥ 24). Different letters above the bars indicate significant differences at the *p* = 0.05 level.

Although DHS could promote cotton embryo growth, it was not detected in substantial amounts at the different ovule/embryo development periods (Figure 2). PHS was the most abundant type of sphingolipid in ovules/embryos and is a direct product of DHS after hydroxylation; we therefore added PHS to in vitro ovule culture and observed embryo changes. The fresh weight of 2 µM PHS-treated embryos after 5 days did not differ significantly from controls, while high concentrations of PHS (6 µM and 20 µM) resulted in 40.3% and 42.0% higher embryo weights, respectively, compared to the control (Figure 4). After 10 days, PHS treatments of 2, 6, and 20 µM produced 47.9%, 93.5%, and 102.1% higher weights, respectively, compared to controls (Figure 4). These results showed that PHS could promote cotton embryo biomass in vitro.

### 3.5. Transcriptomic Analysis of Embryos Treated with PHS

To further explore the molecular mechanisms of PHS regulation of cotton embryo growth, we constructed six cDNA libraries using embryos treated for 10 days with 6 µM PHS for transcriptomic analysis. After sequencing the cDNA libraries, the number of clean reads per library ranged from 44,041,734 to 53,753,302, and over 96.97% of the reads mapped to the upland cotton genome. The percentage with a sequencing quality score > Q30 was at least 93.10%, and the average GC content of the six libraries was 44.34%. These data indicated that the generated reads were of high quality, suitable for differential gene expression analysis. Raw transcriptomic data can be available at SRA database (https://www.ncbi.nlm.nih.gov/sra/PRJNA714828, accessed on 17 March 2021). There were 1983 upregulated and 786 downregulated differentially expressed genes (DEGs) between the control and PHS samples (Table S3). In the top 100 DEGs (FDR < 2.1 × 10^−97^), there were 64 upregulated genes and 36 downregulated genes in the PHS samples (Table S3).

**Figure 5:**
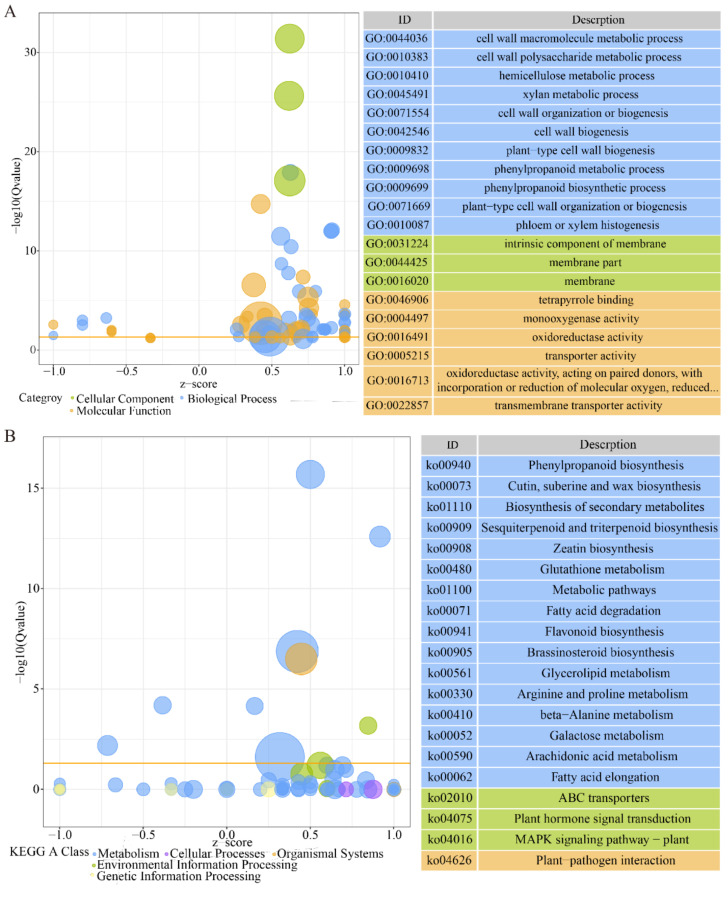
Gene ontology (GO) and Kyoto Encyclopedia of Genes and Genomes (KEGG) enrichment analyses of control and PHS (t18:0, phytosphingosine) samples. (**A**,**B**) The bubble chart of GO (**A**) and KEGG (**B**) enrichment categories (left panel) and the top 20 GO (**A**) and KEGG (**B**) enrichment categories (right panel) in the control and PHS-treated samples. In the bubble chart, the ordinate axes indicate log10 (Q value), and the abscissa axes indicate z-score (the proportion of the difference between the number of up-regulated and down-regulated genes in the total number of DEGs), the bubble sizes mean the difference between the number of up-regulated and down-regulated genes, and the yellow line represents the threshold value of Q value = 0.05. On the right is a list of the top 20 enrichments with Q values. Different colors represent different category or A class and plot separately.

The DEGs were associated with three GO categories (biological process, cellular component, and molecular function). The top 20 GO enrichments included 11 biological processes, 6 molecular functions, and 3 cellular component elements (Table S4). In the biological process category, the most predominant were cell wall macromolecule, cell wall polysaccharide, and hemicellulose metabolic processes. Among the molecular function categories, the most enriched functions among the DEGs were tetrapyrrole binding, monooxygenase activity, and oxidoreductase activity. The cellular component GO terms were those for intrinsic membrane components, membrane parts, and membranes (Figure 5A).

The biological pathways incorporating the genes of interest were identified using KEGG analysis. A total of 16,134 unigenes and 562 DEGs were grouped into 121 known pathways, divided into five categories (Table S5). The top 20 KEGG pathways are shown in Figure 5B; a large number of DEGs were involved in metabolic pathways (252 DEGs, 44.8%), including those for lipids, amino acids, carbohydrates, and terpenoids and polyketides, as well as that for biosynthesis of other secondary metabolites (Figure 5B). Two important signal transduction pathways, those for plant hormone signal transduction (50 DEGs, 8.9%) and plant MAPK signaling (29 DEGs, 5.2%) were also significantly enriched (Figure 5B).

### 3.6. The DEGs of TFs Treated by PHS

**Figure 6:**
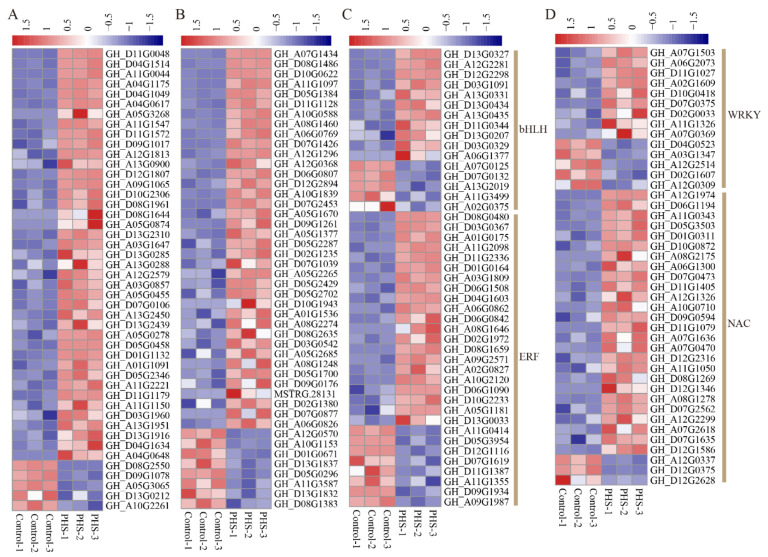
Heat map of differential expression of transcription factors in the control and PHS (t18:0, phytosphingosine)-treated samples cultured for 10 days. (**A**) Zinc finger. (**B**) *MYB*. (**C**) *bHLH* and *ERF*. (**D**) *WRKY* and *NAC*.

Numerous studies have indicated that transcription factors (TFs) participate in plant seed development [22,23,24,25,26,27]. We found that zinc finger, *MYB*, *ERF*, *NAC*, *bHLH*, and *WRKY* were the top six TF families related to embryo growth regulated by PHS, with 46, 46, 29, 29, 16, and 14 member genes present, respectively (Figure 6, Table S3). Among those TFs, identified as differentially expressed, most were upregulated in the PHS samples compared to controls, and the proportions (the percentage of the number of up-regulated TFs to the total number of TFs) were 89.1% (zinc finger), 82.6% (*MYB*), 72.4% (*ERF*), 89.7% (*NAC*), 75.0% (*bHLH*), and 64.3% (*WRKY*) (Figure 6). Furthermore, 10 *LBD*, 9 *BLH*, 9 *MADS*, 7 *GATA*, 5 *ORG*, 5 *NFYA*, 5 *DIVARICATA*, 5 *TCP*, 4 *GRF*, and 4 trihelix TFs were also identified. Of these 63 TFs, only 4 *LBD* and 1 *GATA* were downregulated, while other TFs were upregulated (Figure S1).

### 3.7. The DEGs Related to Lipid Metabolism Treated by PHS

**Figure 7:**
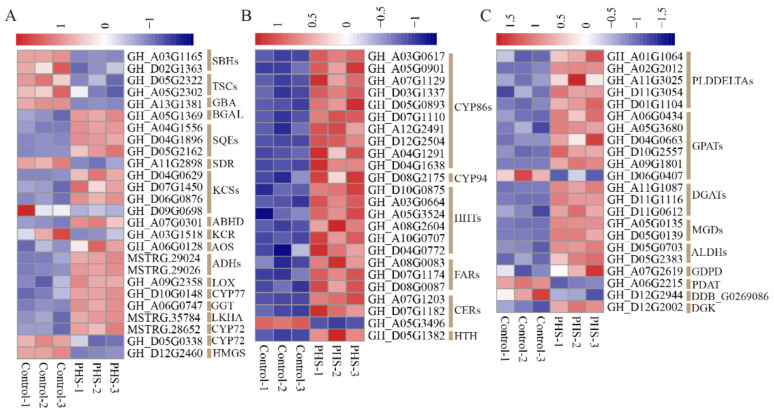
Heat map of differential expression genes (DEGs) related to lipid metabolism in the control and PHS (t18:0, phytosphingosine)-treated samples cultured for 10 days. (**A**) DEGs related to sphingolipid metabolism, steroid biosynthesis, fatty acid biosynthesis and elongation, alpha-linolenic acid and linolenic acid metabolism, arachidonic acid metabolism, and synthesis and degradation of ketone bodies. (**B**) DEGs related to cutin, suberine, and wax biosynthesis. (**C**) DEGs related to glycerolipid and glycerophospholipid metabolism.

Lipids are the main nutrient component of seeds and seed weight is closely related to lipid metabolism [28]. We found that 70 DEGs related to lipid metabolism were altered. Among these genes, there were 26 genes involved in sphingolipid metabolism (2 *SBHs*, 2 *TSCs*, 1 *GBA*, and 1 *BGAL*), steroid biosynthesis (3 *SQEs*), fatty acid biosynthesis and elongation (1 *SDR*, 4 *KCSs*, 1 *ABHD*, and 1 *KCR*), alpha-linolenic acid and linolenic acid metabolism (1 *AOS*, 2 *ADHs*, 1 *LOX* and 1 *CYP77*), arachidonic acid metabolism (1 *GGT*, 1 *LKHA*, and 2 *CYP72s*) and synthesis and degradation of ketone bodies (1 *HMGS*) (Figure 7A). 24 genes were associated with cutin, suberine, and wax biosynthesis (10 *CYP86s*, 1 *CYP94*, 6 *HHTs*, 3 *FARs*, 3 *CERs*, and 1 *HTH*) (Figure 7B). Another 22 genes were involved in glycerolipid and glycerophospholipid metabolism (5 *PLDDELTAs*, 6 *GPATs*, 3 *DGATs*, 2 *MGDs*, 2 *ALDHs*, 1 *GDPD*, 1*PDAT*, 1 *DDB_G0269086*, and 1 *DGK*) (Figure 7C). Of those genes, only 14 were downregulated. Most DEGs related to sphingolipid metabolism were downregulated, while almost all DEGs related to cutin, suberine, and wax biosynthesis (except 1 *HTH*) were upregulated.

### 3.8. The DEGs Related to Plant Hormones Treated by PHS

**Figure 8:**
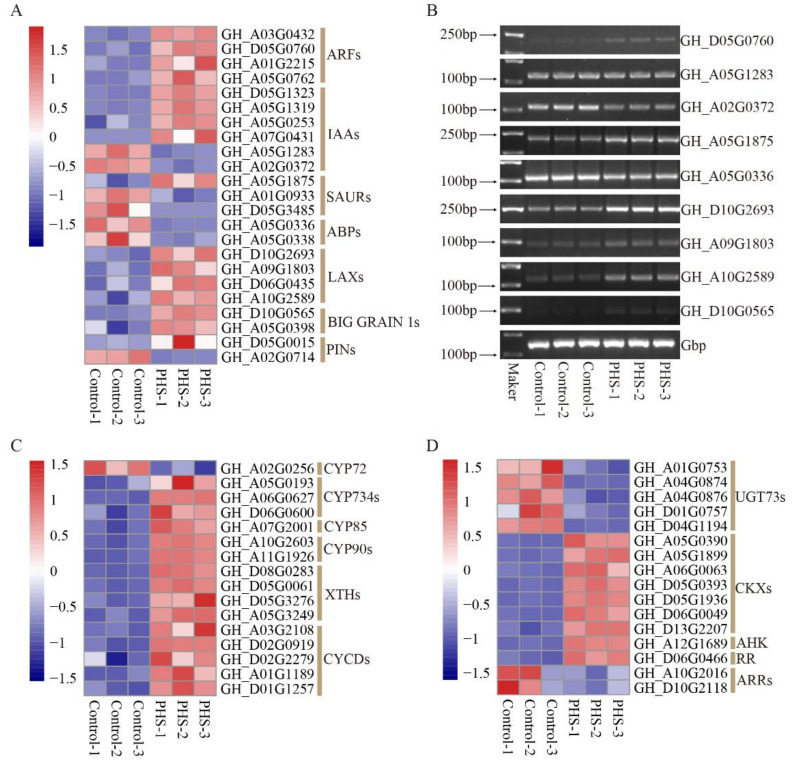
Involvement of differentially expressed genes (DEGs) related to plant hormones in PHS (t18:0, phytosphingosine)-related promotion of embryo growth. (**A**) Heat map of auxin signaling pathway gene expression in the control and PHS-treated samples after culture for 10 days. (**B**) Semi-quantitative PCR of auxin signaling pathway genes in the same control and PHS samples. DL2000 DNA marker (left). Amplified genes of interest (right). (**C**,**D**) Heat map of brassinosteroid (**C**) and zeatin (**D**) biosynthesis and signal transduction pathway gene expression in the control and PHS-treated samples after culture for 10 days.

Auxin regulates many of the processes of plant growth and development, and plays an important role in seed development. In this study, auxin-related gene expression was analyzed to uncover how the auxin signaling pathway was involved in PHS-related promotion of embryo growth. In total, 23 auxin signaling pathway-related genes were among those affected by PHS, including 4 auxin response factors (*ARF*s), 9 auxin-responsive proteins (6 *IAA*s and 3 *SAUR*s), 2 auxin-binding proteins (*ABP*s), 4 auxin transporter-like proteins (*LAX*s), 2 auxin transport proteins (*BIG GRAIN 1*s), and 2 auxin efflux carriers (*PIN*s) (Figure 8A). Among these genes, all *ARF*s, *LAX*s, *BIG GRAIN 1*s, and most *IAA*s were upregulated, while all *ABP*s and most *SAUR*s were downregulated (Figure 8A). Thereafter, we randomly selected several genes to verify their transcription levels by means of semi-quantitative PCR. Our results showed that the transcription of each gene was consistent with the changes detected by RNA-seq (Figure 8B).

We also found that brassinosteroid (BR) and zeatin biosynthesis and signal transduction pathway genes were altered. 7 BR biosynthesis (1 *CYP72*, 3 *CYP734s*, 1 *CYP85*, and 2 *CYP90s*) and 9 BR-responsive proteins (4 *XTHs* and 5 *CYCDs*) related genes were identified (Figure 8C). There were 12 genes involved in zeatin biosynthesis (5 *UGT73s* and 7 *CKXs*) and 4 genes involved in zeatin signal transduction pathway (1 *AHK*, 1 *PR*, and 2 *ARRs*) (Figure 8D). Among DEGs related to BR, only 1 *CYP72* were downregulated, while all *UGT73s* and *ARRs* related to zeatin were downregulated.

## 4. Discussion

Sphingolipids are not only the main structural components of biomembrane, but also important bioactive molecules that participate in various signal transduction pathways. They play an important role in plant growth, development, and stress response [29,30,31,32]. In a previous study, we found that the sphingolipid synthesis inhibitor FB1 can seriously interfere with cotton fiber elongation [6]. Here, we investigated whether FB1 can also affect cotton embryo growth (Figure 1), finding that sphingolipid homeostasis does indeed play an important role not only in cotton fiber elongation, but also in embryo growth. Although we identified a large number of sphingolipids and proteins affected by FB1 in cotton fibers and embryos, the functions and regulatory mechanisms of individual sphingolipids in cotton are still unclear.

In this study, we examined sphingolipid content in ovules/embryos from field-grown plants at different developmental stages. Concordant with the results for sphingolipids in fibers and embryos cultured in vitro [6], LCBs were the most abundant sphingolipids (Figure 3). In Arabidopsis seedlings, the most abundant sphingolipids are GIPCs, while LCBs account for only a small fraction [10]. These results suggest that sphingolipid composition varies greatly among different species, and that LCBs may play a special role in cotton embryo growth.

FB1 and myriocin are sphingolipid inhibitors that alter sphingolipid content [33]. FB1 results in an increase in simple sphingolipids (LCBs) and a decrease in complex sphingolipids in Arabidopsis [33]. This condition also occurred in FB1 treated cotton fibers and embryos [6]. Myriocin can inhibit SPT enzyme activity and reduce LCB and GIPC content [33,34]. In this study, we found that both FB1 and myriocin can reduce the fresh weight of cotton embryos, while LCBs (DHS and PHS) can increase it (Figure 1, Figure 2 and Figure 4). Although FB1 can increase the LCB content, it still decreases embryo fresh weight. Therefore, we speculate that LCBs increase cotton embryo biomass by influencing the downstream sphingolipid content, especially that of the complex GIPC sphingolipids. Studies of Arabidopsis sphingolipid-related mutants have gradually revealed the functions of some sphingolipid synthase genes in plant development. It is possible to identify cotton sphingolipid synthesis genes through multiple sequence alignment. These sphingolipid synthase proteins in cotton may have conserved functions. Future studies could investigate sphingolipid function in cotton embryos and improve cotton seed traits by modifying the expression levels of sphingolipid synthesis genes through genetic transformation.

Larger seeds provide more nutrients for seed germination and can also improve seed resistance to environmental stress. Seed development is regulated by a complex gene network. Given that PHS was shown to increase the fresh weight of cotton embryos, we performed a global transcriptomic analysis in order to reveal the associated regulatory mechanism. We identified a great number of DEGs in the control and PHS samples. In a previous study, 20 peroxidases (*POD*s) were upregulated in FB1 treated cotton fibers and embryos [6]. This study also found 25 upregulated *POD*s in the PHS-treated samples (Table S3). We speculate that *POD*s may be activated by LCBs rather than by complex sphingolipids. *POD*s play an important role in plant development [35]. LCBs may mediate *POD*-based regulation of plant growth processes apart from affecting cotton seed development, because cotton embryos became smaller under both the FB1 and myriocin treatments.

As lipid rafts contain a variety of signaling molecules, they can participate in many signal transduction pathways. Sphingolipids, as lipid raft components, may affect raft activity, thereby affecting signal transduction. Meanwhile, the activity of TFs is activated or inhibited, leading to gene expression changes. We found a large number of differentially expressed TFs, most of which were upregulated in PHS-treated samples, which could explain why the number of upregulated genes (1983) was much higher than that of downregulated genes (786) in these samples. TFs are involved in every aspect of plant growth and development, including seed development. The *GS2* QTL, coding for OsGRF4 in rice, promotes grain size by enlarging glume cells [23]. The *class D* genes of the *MADS-box* family are crucial in regulating ovule development [22,24]. *NARSL* and *NARS2* encode NAC TFs that are responsible for regulating the growth and degeneration of ovule tepals in Arabidopsis [25]. The *bHLH* transcription factor *RGE1* gene in the endosperm plays an important role in controlling embryo growth in Arabidopsis [36]. *TTG2* and *MINI3* are *WRKY* TFs that modulate seed size [37,38,39]. These important transcription factors that regulate seed development are also present in our transcriptome data. In particular, some of the pathways that regulate seed size were altered by PHS. For example, *IKU2* encodes a leucine-rich repeat (LRR) kinase and shares a pathway of seed development with *MINI3*. We also found 19 upregulated *LRR* kinase genes in PHS samples (Table S3). We speculate that the *MINI3*-*IKU2* pathway is conserved in cotton embryo development.

Numerous studies have shown a correlation between lipid metabolism and seed size or weight. For example, miRNA167A promotes seed size and decreases α-linolenic acid content in *Camelina sativa* [40]. Silencing of *GmFAD3* leads to larger seeds and reduces linolenic acid (18:3) in soybean [41]. *TT2* mutation decreases seed weight and increases fatty acid content in Arabidopsis [42]. In this study, 70 DEGs were related to various lipid metabolism processes, and most of them were upregulated, in particular, cutin, suberine, and wax biosynthesis pathway. In tomato, overexpression of *SlKLUH* confers an increase in seed weight and lower expression of *SlKLUH* was associated with increased expression of genes involved in lipid metabolism, including cutin synthesis and transport pathway, and fatty acid elongation and wax biosynthesis pathway [28]. These results indicate lipid metabolism and seed weight have a complicated relationship. In addition, we found that 4 DEGs (2 *SBHs* and 2 *TSCs*) related to sphingosine biosynthesis were downregulated. This suggests that PHS may negatively feedback sphingosine synthesis.

Auxin response is mediated by *ARF* transcription factors, which transcriptionally regulate the downstream auxin genes [43]. In Arabidopsis, *arf2* mutants have larger seeds than those of wildtype plants [43]. In *Brassica napus*, *ARF18* acts as an inhibitor of the auxin response and limits cell elongation to control seed size [44]. Overexpression of the *JcARF19* gene in *Jatropha curcas* resulted in enlarged seeds in *Jatropha curcas*; a similar effect was seen in Arabidopsis [45]. The auxin transport protein BIG GRAIN 1 can promote grain size in both rice and maize [46,47]. In this study, we also identified transcript alterations associated with the auxin signaling pathway, including auxin response, binding, and transport, which have been reported to be involved in seed development. Among them, 4 *ARF*s and 2 *BIG GRAIN 1*s were upregulated in PHS-treated samples. Several studies have shown that brassinosteroid (BR) related genes are closely related to seed weight. *DWF4*, encoding a cytochrome P450 enzyme (CYP90B1), increases seed weight in Arabidopsis [48], rice [49], and *Brassica napus* [50]. D-type cyclins (CYCD) are promotes cell division and the expression of *CYCD3* could be induced by BR in Arabidopsis [51]. Activation of *CYCD7;1* leads to larger seeds in Arabidopsis [52]. Here, we found 6 *CYPs* related to BR biosynthesis and 5 *CYCDs* involved in the BR signal transduction pathway were upregulated in PHS-treated samples. PHS may mediate cotton embryo development through these genes; future study of their functions may support improvement of cotton seed traits.

## 5. Conclusions

Our results reveal important sphingolipid molecules and gene regulatory networks involved in cotton embryo growth. Both the sphingolipid synthesis inhibitor FB1 and the SPT enzyme inhibitor myriocin can reduce cotton embryo fresh weight. PHS were the most abundant sphingolipids at different developmental stages of cotton ovules/embryos and increased embryo fresh weight. These findings not only provide key metabolites for consideration in seed improvement, but also provide a strong rationale for the use of RNA-Seq analysis in future studies. Through the analysis of DEGs, we found that a large number of TFs were upregulated, including *WRKY*, *NAC*, *MADS-box*, and *GRF*, all of which have been reported to be involved in seed development. The lipid metabolism and plant hormones (auxin, brassinosteroid, and zeatin) related genes were also altered and most of them were upregulated. These DEGs may be targeted to improve seed biomass in the future.

## Data Availability

Publicly available datasets were analyzed in this study. This data can be found here: https://www.ncbi.nlm.nih.gov/sra/PRJNA714828 (accessed on 7 June 2020).

## Supplementary Materials

The following are available online at https://www.mdpi.com/article/10.3390/biom11040525/s1, Figure S1: Heat map of differential expression of TFs in control and PHS samples cultured 10 days; Table S1: Primer sequences for semi-quantitative PCR; Table S2: Individual sphingolipid content of ovules/embryos at five different developmental stages; Table S3: The list of all DEGs in control and the PHS samples; Table S4: GO analysis of all DEGs in the control and the PHS samples; and Table S5: KEGG analysis of all DEGs in the control and the PHS samples.

## References

[1] Lynch D.V., Dunn T.M. (2004). An introduction to plant sphingolipids and a review of recent advances in understanding their metabolism and function. New Phytol..

[2] Markham J.E., Lynch D.V., Napier J.A., Dunn T.M., Cahoon E.B. (2013). Plant sphingolipids: Function follows form. Curr. Opin. Plant Biol..

[3] Chen M., Markham J.E., Dietrich C.R., Jaworski J.G., Cahoon E.B. (2008). Sphingolipid long-chain base hydroxylation is important for growth and regulation of sphingolipid content and composition in Arabidopsis. Plant Cell.

[4] Chen M., Markham J.E., Cahoon E.B. (2012). Sphingolipid delta 8 unsaturation is important for glucosylceramide biosynthesis and low-temperature performance in Arabidopsis. Plant J..

[5] Markham J.E., Molino D., Gissot L., Bellec Y., Hematy K., Marion J., Belcram K., Palauqui J.C., Satiat-JeuneMaitre B., Faure J.D. (2011). Sphingolipids Containing Very-Long-Chain Fatty Acids Define a Secretory Pathway for Specific Polar Plasma Membrane Protein Targeting in Arabidopsis. Plant Cell.

[6] Wang L., Liu C., Liu Y.J., Luo M. (2020). Fumonisin B1-Induced Changes in Cotton Fiber Elongation Revealed by Sphingolipidomics and Proteomics. Biomolecules.

[7] Marques J.T., Marinho H.S., de Almeida R.F.M. (2018). Sphingolipid hydroxylation in mammals, yeast and plants—An integrated view. Prog. Lipid Res..

[8] Beasley C.A., Ting I.P. (1973). The effects of plant growth substances on in vitro fiber development from fertilized cotton ovules. Am. J. Bot..

[9] Welti R., Li W.Q., Li M.Y., Sang Y.M., Biesiada H., Zhou H.E., Rajashekar C.B., Williams T.D., Wang X.M. (2002). Profiling membrane lipids in plant stress responses—Role of phospholipase D alpha in freezing-induced lipid changes in Arabidopsis. J. Biol. Chem..

[10] Huang D.Q., Sun Y.B., Ma Z.M., Ke M.Y., Cui Y., Chen Z.C., Chen C.F., Ji C.Y., Tran T.M., Yang L. (2020). Salicylic acid-mediated plasmodesmal closure via Remorin-dependent lipid organization. Proc. Natl. Acad. Sci. USA.

[11] Markham J.E., Jaworski J.G. (2007). Rapid measurement of sphingolipids from Arabidopsis thaliana by reversed-phase high-performance liquid chromatography coupled to electrospray ionization tandem mass spectrometry. Rapid Commun. Mass Spectrom..

[12] Chen S.F., Zhou Y.Q., Chen Y.R., Gu J. (2018). Fastp: An ultra-fast all-in-one FASTQ preprocessor. Bioinformatics.

[13] Langmead B., Salzberg S.L. (2012). Fast gapped-read alignment with Bowtie. Nat. Methods.

[14] Kim D., Landmead B., Salzberg S.L. (2015). HISAT: A fast spliced aligner with low memory requirements. Nat. Methods.

[15] Hu Y., Chen J.D., Fang L., Zhang Z.Y., Ma W., Niu Y.C., Ju L.Z., Deng J.Q., Zhao T., Lian J.M. (2019). Gossypium barbadense and Gossypium hirsutum genomes provide insights into the origin and evolution of allotetraploid cotton. Nat. Genet..

[16] Pertea M., Pertea G.M., Antonescu C.M., Chang T.C., Mendell J.T., Salzberg S.L. (2015). StringTie enables improved reconstruction of a transcriptome from RNA-seq reads. Nat. Biotechnol..

[17] Pertea M., Kim D., Pertea G.M., Leek J.T., Salzberg S.L. (2016). Transcript-level expression analysis of RNA-seq experiments with HISAT, StringTie and Ballgown. Nat. Protoc..

[18] Li B., Dewey C.N. (2011). RSEM: Accurate transcript quantification from RNA-Seq data with or without a reference genome. BMC Bioinform..

[19] Love M.I., Huber W., Anders S. (2014). Moderated estimation of fold change and dispersion for RNA-seq data with Deseq2. Genome Biol..

[20] Ashburner M., Ball C.A., Blake J.A., Botstein D., Butler H., Cherry J.M., Davis A.P., Dolinski K., Dwight S.S., Eppig J.T. (2000). Gene Ontology: Tool for the unification of biology. Nat. Genet..

[21] Kanehisa M., Goto S. (2000). KEGG: Kyoto Encyclopedia of Genes and Genomes. Nucleic Acids Res..

[22] Angenent G.C., Franken J., Busscher M., Vandijken A., Vanwent J.L., Dons H.J.M., Vantunen A.J. (1995). A Novel Class of Mads Box Genes Is Involved in Ovule Development in Petunia. Plant Cell.

[23] Duan P.G., Ni S., Wang J.M., Zhang B.L., Xu R., Wang Y.X., Chen H.Q., Zhu X.D., Li Y.H. (2016). Regulation of OsGRF4 by OsmiR396 controls grain size and yield in rice. Nat. Plants.

[24] Ehlers K., Bhide A.S., Tekleyohans D.G., Wittkop B., Snowdon R.J., Becker A. (2016). The MADS Box Genes ABS, SHP1, and SHP2 Are Essential for the Coordination of Cell Divisions in Ovule and Seed Coat Development and for Endosperm Formation in Arabidopsis thaliana. PLoS ONE.

[25] Kunieda T., Mitsuda N., Ohme-Takagi M., Takeda S., Aida M., Tasaka M., Kondo M., Nishimura M., Hara-Nishimura I. (2008). NAC Family Proteins NARS1/NAC2 and NARS2/NAM in the Outer Integument Regulate Embryogenesis in Arabidopsis. Plant Cell.

[26] Ohto M., Fischer R.L., Goldberg R.B., Nakamura K., Harada J.J. (2005). Control of seed mass by Apetala2. Proc. Natl. Acad. Sci. USA.

[27] Sun X.D., Shantharaj D., Kang X.J., Ni M. (2010). Transcriptional and hormonal signaling control of Arabidopsis seed development. Curr. Opin. Plant Biol..

[28] Li Q., Chakrabarti M., Taitano N., Okazaki Y., Saito K., Al-Abdallat A., van der Knaap E. (2021). Differential expression of SlKLUH controlling fruit and seed weight is associated with changes in lipid metabolism and photosynthesis-related genes. J. Exp. Bot..

[29] Liang H., Yao N., Song L.T., Luo S., Lu H., Greenberg L.T. (2003). Ceramides modulate programmed cell death in plants. Genes Dev..

[30] Bi F.C., Liu Z., Wu J.X., Liang H., Xi X.L., Fang C., Sun T.J., Yin J., Dai G.Y., Rong C. (2014). Loss of Ceramide Kinase in Arabidopsis Impairs Defenses and Promotes Ceramide Accumulation and Mitochondrial H2O2 Bursts. Plant Cell.

[31] Wu J.X., Li J., Liu Z., Yin J., Chang Z.Y., Rong C., Wu J.L., Bi F.C., Yao N. (2015). The Arabidopsis ceramidase AtACER functions in disease resistance and salt tolerance. Plant J..

[32] Zheng P., Wu J.X., Sahu S.K., Zeng H.Y., Huang L.Q., Liu Z., Xiao S., Yao N. (2018). Loss of alkaline ceramidase inhibits autophagy in Arabidopsis and plays an important role during environmental stress response. Plant Cell Environ..

[33] Iswanto A.B.B., Shon J.C., Liu K.H., Vu M.H., Kumar R., Kim J.Y. (2020). Sphingolipids Modulate Secretion of Glycosylphosphatidylinositol-Anchored Plasmodesmata Proteins and Callose Deposition. Plant Physiol..

[34] Hanada K. (2004). Serine palmitoyltransferase, a key enzyme of sphingolipid metabolism. Biochim. Biophys. Acta Mol. Cell Biol. Lipids.

[35] Francoz E., Ranocha P., Nguyen-Kim H., Jamet E., Burlat V., Dunand C. (2015). Roles of cell wall peroxidases in plant development. Phytochemistry.

[36] Kondou Y., Nakazawa M., Kawashima M., Ichikawa T., Yoshizumi T., Suzuki K., Ishikawa A., Koshi T., Matsui R., Muto S. (2008). Retarded Growth of Embryo1, a new basic helix-loop-helix protein, expresses in endosperm to control embryo growth. Plant Physiol..

[37] Garcia D., Fitz Gerald J.N., Berger F. (2005). Maternal control of integument cell elongation and zygotic control of endosperm growth are coordinated to determine seed size in Arabidopsis. Plant Cell.

[38] Wang A.H., Garcia D., Zhang H.Y., Feng K., Chaudhury A., Berger F., Peacock W.J., Dennis E.S., Luo M. (2010). The VQ motif protein IKU1 regulates endosperm growth and seed size in Arabidopsis. Plant J..

[39] Luo M., Dennis E.S., Berger F., Peacock W.J., Chaudhury A. (2005). MINISEED3 (MINI3), a WRKY family gene, and HAIKU2 (IKU2), a leucine-rich repeat (LRR) KINASE gene, are regulators of seed size in Arabidopsis. Proc. Natl. Acad. Sci. USA.

[40] Na G., Mu X.P., Grabowski P., Schmutz J., Lu C.F. (2019). Enhancing microRNA167A expression in seed decreases the alpha-linolenic acid content and increases seed size in Camelina sativa. Plant J..

[41] Singh A.K., Fu D.Q., El-Habbak M., Navarre D., Ghabrial S., Kachroo A. (2011). Silencing Genes Encoding Omega-3 Fatty Acid Desaturase Alters Seed Size and Accumulation of Bean pod mottle virus in Soybean. Mol. Plant Microbe. Interact..

[42] Chen M.X., Wang Z., Zhu Y.N., Li Z.L., Hussain N., Xuan L.J., Guo W.L., Zhang G.P., Jiang L.X. (2012). The Effect of Transparent Testa2 on Seed Fatty Acid Biosynthesis and Tolerance to Environmental Stresses during Young Seedling Establishment in Arabidopsis. Plant Physiol..

[43] Schruff M.C., Spielman M., Tiwari S., Adams S., Fenby N., Scott R.J. (2006). The AUXIN RESPONSE FACTOR 2 gene of Arabidopsis links auxin signalling, cell division, and the size of seeds and other organs. Development.

[44] Liu J., Hua W., Hu Z.Y., Yang H.L., Zhang L., Li R.J., Deng L.B., Sun X.C., Wang X.F., Wang H.Z. (2015). Natural variation in ARF18 gene simultaneously affects seed weight and silique length in polyploid rapeseed. Proc. Natl. Acad. Sci. USA.

[45] Sun Y.W., Wang C.M., Wang N., Jiang X.Y., Mao H.Z., Zhu C.X., Wen F.J., Wang X.H., Lu Z.J., Yue G.H. (2017). Manipulation of Auxin Response Factor 19 affects seed size in the woody perennial Jatropha curcas. Sci. Rep..

[46] Liu L.C., Tong H.N., Xiao Y.H., Che R.H., Xu F., Hu B., Liang C.Z., Chu J.F., Li J.Y., Chu C.C. (2015). Activation of Big Grain1 significantly improves grain size by regulating auxin transport in rice. Proc. Natl. Acad. Sci. USA.

[47] Simmons C.R., Weers B.P., Reimann K.S., Abbitt S.E., Frank M.J., Wang W.Y., Wu J.R., Shen B., Habben J.E. (2020). Maize BIG GRAIN1 homolog overexpression increases maize grain yield. Plant Biotechnol. J..

[48] Choe S., Fujioka S., Noguchi T., Takatsuto S., Yoshida S., Feldmann K.A. (2001). Overexpression of DWARF4 in the brassinosteroid biosynthetic pathway results in increased vegetative growth and seed yield in Arabidopsis. Plant J..

[49] Wu C.Y., Trieu A., Radhakrishnan P., Kwok S.F., Harris S., Zhang K., Wang J.L., Wan J.M., Zhai H.Q., Takatsuto S. (2008). Brassinosteroids regulate grain filling in rice. Plant Cell.

[50] Sahni S., Prasad B.D., Liu Q., Grbic V., Sharpe A., Singh S.P., Krishna P. (2016). Overexpression of the brassinosteroid biosynthetic gene DWF4 in Brassica napus simultaneously increases seed yield and stress tolerance. Sci. Rep..

[51] Hu Y.X., Bao F., Li J.Y. (2000). Promotive effect of brassinosteroids on cell division involves a distinct CycD3-induction pathway in Arabidopsis. Plant J..

[52] Sornay E., Forzani C., Forero-Vargas M., Dewitte W., Murray J.A.H. (2015). Activation of CYCD7;1 in the central cell and early endosperm overcomes cell-cycle arrest in the Arabidopsis female gametophyte, and promotes early endosperm and embryo development. Plant J..

