# Supplementary figures and images for: Sphingosine Promotes Embryo Biomass in Upland Cotton: A Biochemical and Transcriptomic Analysis
Source: Biomolecules. 2021 Apr 1;11(4):525. doi: 10.3390/biom11040525 (PMC8065874; doi:10.3390/biom11040525)

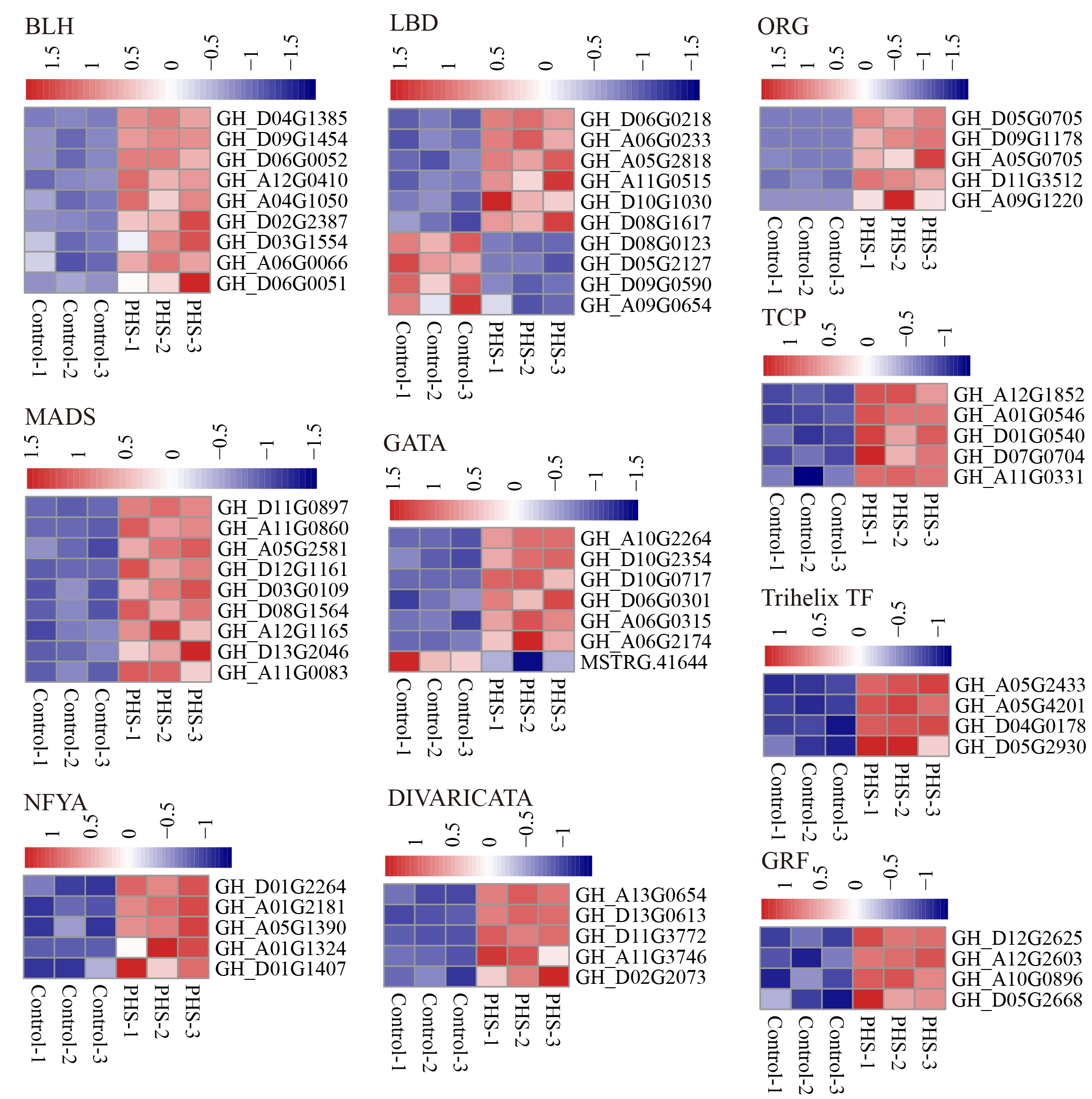

Supplement: Supplementary file 1 [file biomolecules-11-00525-s001.zip › supplementary files/Fig S1.tif]
